# Editorial: Antiviral immune response in fish and shellfish

**DOI:** 10.3389/fimmu.2023.1155538

**Published:** 2023-03-28

**Authors:** M. Ortega-Villaizan, L. Mercado, V. Chico

**Affiliations:** ^1^ Instituto de Investigación, Desarrollo e Innovación en Biotecnología Sanitaria de Elche (IDiBE), Universidad Miguel Hernández (IDiBE-UMH), Elche, Spain; ^2^ Instituto de Biología, Pontificia Universidad Católica de Valparaíso, Valparaíso, Chile

**Keywords:** fish, shellfish, antiviral, immune response, virus, bacteria, innate immunity

Innate immunity constitutes the main defense mechanism in fish and shellfish, which is the most antique and efficient system to protect organisms against microbial infections. In addition, lower vertebrates have less evolved adaptive immune responses compared to higher vertebrates, and therefore, rely on innate immune defenses for the control of pathogens (1). Nonetheless, it is worth noting that the contribution of both innate and adaptive immune responses leads to global protection against pathogens in the organism.

The objective of this Research Topic is to reflect the significant advances in the study of the immune response against viral infections in fish. This topic contributes to identifying new mechanisms of response to infections and new effector molecules against viral pathogens. In addition, these studies provide a further understanding of the resistance of lower vertebrates to pathogens infection as well as to the prevention of exacerbated immune responses.

The contributing articles collected in this Research Topic are grouped into the following proposed sections related to the antiviral defense in fish:

## 1 Antiviral mechanisms involved in innate immunity and cellular response to viral infections in fish

The first mechanism of response to infection is the innate immune response which comprises a set of cells and molecules that are involved in the body’s defense against infections.

In this section, we can find the advances in the understanding of the defense strategies in the Atlantic salmon against viral infections through a meta-analysis based on microarray transcriptomic analysis. Krasnov et al. were able to distinguish between pathogen-specific responses from stress and general inflammation. They found that viral responsive genes were similar to different stressors such as DNA or RNA viruses, to the injection of bacterial DNA (plasmid), and the exposure to PAMPs (CpG and gardiquimod), but they responded to inflammation and bacteria with quite a low sensitivity.

Vallejos-Vidal et al. revised the immune response mechanisms of one of the most pathogenic viruses, which affects farmed Atlantic salmon, the piscine orthoreovirus (PRV). They revised how an experimental infection of PRV triggers the antiviral immune response in salmonid RBCs. In addition, they showed how PRV elicited a Th1-type response and a positive regulatory effect on the CTL-mediated immune responses. The immune mechanisms related to the phenotype of permanently PRV-infected fish and their effects on the progression of secondary infection were also reviewed.

Malik et al. showed the problem around melanized focal changes “black spots” in the white skeletal muscle of farmed Atlantic salmons infected with PRV-1. The first symptoms observed, which were the “red spots”, were featured by hemorrhages and acute inflammation that progressed into chronic inflammation developing the “black spots” in the muscle of salmons. Although the etiology of the spots is unknown, in this study, the authors found in both the red and black spots the presence of macrophages and melano-macrophages associated with abundant PRV-1. Here the authors studied the polarization status of the macrophages and cell-mediated immune responses in both PRV-1-infected and non-infected fish. Altogether, in this study, the authors suggested that PRV-1 could contribute to the pro-inflammatory environment that is essential for the pathogenesis of the spots.

Finally, in this section, Jia et al. shed light on the mechanisms related to the immune response involved in both sensitivity and resistance against cyprinid herpesvirus-3 (CyHV3) in common carp. The authors demonstrated, with a transcriptomic analysis, that specific innate immune mechanisms which included phagocytosis, autophagy, cytotoxicity, and virus blockage by lectins and mucins like mucin 3 (MUC3) were the cause of a marked resistance to CyHV-3 infection in a breeding carp strain.

## 2 Antiviral determinants and molecules responsible for defense mechanisms

Pathogens are able to trigger the immune response in the host through the activation of a huge variety of determinants or molecules responsible for the defense mechanisms. In this section, the authors identified two antiviral molecules and studied their role in the immune response of fish.

Mou et al. identified different antiviral mechanisms for two viperin homologs (Cgviperin-A and Cgviperin-B) in the auto-allo-hexaploid gibel carp strain against crucian carp (*Carasius auratus*) herpesvirus (CaHV). The C-terminal domain of CgViperin-A and CgViperin-B were found to interact with a negative herpesvirus regulator of host interferon (IFN) production, the CaHV open reading frame 46 right (ORF46R) protein, and to develop the proteasomal degradation of ORF46R *via* reducing K63-linked ubiquitination. CgViperin-B was also found to mediate ORF46R degradation through autophagosomes. Their findings also clarified the different antiviral mechanisms of the duplicated viperin homologs in polyploid fish, which elucidated the evolution of teleost duplicated genes.

Liu et al. showed how the toll-interleukin receptor (TIR)-domain-containing adapter-inducing interferon-β (TRIF), an essential adaptor downstream of Toll-like receptor signaling, played an important role in the innate immune response. The authors showed how common carp TRIF inhibited the replication of spring viremia carp virus (SVCV) in epithelioma papulosum cyprini (EPC) cells. In addition, the authors showed that TRIF increased under *Aeromonas hydrophila* and poly (I:C) stimulation *in vivo* and under poly (I:C), lipopolysaccharide, flagellin, peptidoglycan, and Pam3CSK4 stimulation *in vitro*. In summary, this study indicated that TRIF plays an important role in the innate immune responses of common carp against viral and bacterial infections.

## 3 Molecular regulation of innate immune responses upon viral infection

A balanced immune response can protect the organism from pathogens, but an exacerbated response can impair the immune homeostasis, leading to uncontrolled inflammation or pathogen invasion. In this section, the molecular regulation of some innate immune responses against viral infection was reviewed.

MicroRNAs (miRNAs) are molecules that are extensively involved in the regulatory systems of inflammation and immune responses in mammals. However, the regulatory pathway of miRNA-mediated immune responses is not well understood in lower vertebrates. In this section, the authors showed that different miRNAs could play an adverse role in the *Miiuy croaker* antimicrobial immunity. Gao et al. showed that pathogens such as rhabdovirus and bacteria up-regulated the expression of miRNAs, showing that an up-regulation of miR-2187 was able to reduce the production of antiviral genes and inflammatory factors through targeting TNF receptor-associated factor 6 (TRAF6), therefore, avoiding an extreme inflammatory response. On the other hand, Sun et al. showed that miR-181b-2 and miR-21-1 modulated antiviral and antibacterial immunity by means of the TRIF-mediated nuclear factor-κB (NF-κB) and interferon regulatory factor 3 (IRF3) signaling pathways.

In the same way, Li et al. described the role of miR-124 in the cellular immune response of *Epinephelus coioides* by Singapore grouper iridovirus (SGIV) infection (entry and replication). The authors showed a significant up-regulation of the miR-124 expression after SGIV infection. Their results suggested that *E. coioides* miR-124 increased viral replication and negatively modulated the host immune response by targeting Jun N-terminal kinases 3 (JNK3)/p38α MAPKinasa. Their results broaden the knowledge of the host immune interactions with viruses.

Metabolites are also known to regulate the immune response and the susceptibility to pathogen infections. He et al. performed metabolome studies of Grass carp infected with GCRV and showed that, after viral infection, most metabolites increased in three-year-old fish and decreased in five-month-old fish. In addition, those differentially expressed metabolites presented antiviral effects both *in vivo* and *in vitro*. In summary, the authors concluded that the age-dependent viral susceptibility in grass carp depended on the immune system and metabolism of the host.

In summary, this Research Topic compiles recent developments and research in relation to the importance of innate immunity in antiviral defense in fish. Our objective was to increase the interest of research communities in these areas of research and direct this knowledge into new perspectives and strategies related to antiviral defense across aquatic organisms.

## Author contributions

VC and MO-V wrote the manuscript with contributions from other authors. VC, MO-V and LM contributed to conception of the Research Topic. All authors contributed to the article and approved the submitted version.
